# Safety of autologous freshly expanded mesenchymal stromal cells for the treatment of graft-versus-host disease

**DOI:** 10.3389/fimmu.2022.959658

**Published:** 2022-09-14

**Authors:** Elizabeth Stenger, Cynthia R. Giver, Amelia Langston, Daniel Kota, Pankoj Kumar Das, Raghavan Chinnadurai, Jacques Galipeau, Edmund K. Waller, Muna Qayed

**Affiliations:** ^1^ Aflac Cancer and Blood Disorders Center, Children’s Healthcare of Atlanta, Emory University, Atlanta, GA, United States; ^2^ Bone Marrow and Stem Cell Transplant Center, Winship Cancer Institute, Emory University, Atlanta, GA, United States; ^3^ Department of Biomedical Sciences, Mercer University School of Medicine, Savannah, GA, United States; ^4^ Department of Medicine and Carbone Cancer Center, University of Wisconsin in Madison, Madison, WI, United States

**Keywords:** Mesechymal stromal cell, acute graft versus host disease (aGVHD), chronic graft versus host disease (GVHD), allogeneic transplant of haematopoietic stem cells, Hematologic malignancy

## Abstract

Despite the curative potential of hematopoietic cell transplantation (HCT) for hematologic malignancies, graft-versus-host disease (GVHD) remains a substantial cause of morbidity and mortality, particularly if treatment is refractory. Treatment with additional immunosuppression including steroids often leads to opportunistic infections and organ dysfunction. Novel therapies are greatly needed, specifically ones that lead to responses in treatment-refractory patients and are better tolerated. Mesenchymal stromal cells (MSCs) are non-hematopoietic tolerogenic cells present in normal bone marrow (BM), which can be expanded *ex vivo* to therapeutic doses. Their safety and efficacy have been assessed in inflammatory disorders including GVHD, but heterogeneity in clinical responses has led some to examine MSC manufacturing and administration procedures, which may impact *in vivo* efficacy. We hypothesized that autologous, early-passage, and culture-recovered (after freeze and thaw) MSCs would be safe and may have superior efficacy. In this phase I single-center trial, we assessed MSC safety and early efficacy of an escalating number of doses (2 × 10^6^/kg doses; dose level 1, single dose; dose level 2, two weekly doses; dose level 3, four weekly doses) in patients aged ≥12 years with treatment-refractory acute or chronic GVHD. Eleven enrolled patients received some or all planned MSC infusions, with a median age at enrollment of 37 years. The most common primary HCT indication was leukemia, and the median time from HCT to first MSC infusion was 2.6 years. MSC infusion was well tolerated, with all severe adverse events expected and determined to be unlikely or definitely not related to the study. Thus, no dose-limiting toxicities occurred in the three dose levels. Three of four patients with acute GVHD (or overlap with acute features) had responses seen at any timepoint, ranging from partial to complete. In those with a chronic GVHD indication (n = 7), an overall response at 3 months was partial in five, stable in one, and progressive in one. No appreciable differences were seen between dose levels in peripheral blood lymphocyte subsets. In conclusion, autologous and culture-recovered MSCs were safe in the setting of refractory GVHD following HCT for hematologic malignancy, and clinical responses were most notable in patients with acute GVHD.

## Introduction

Hematopoietic cell transplantation (HCT) is the only curative option for many hematologic malignancies, in which healthy donor hematopoietic stem cells (HSCs) are infused following typically high doses of chemotherapy (1). One of the main complications of HCT is graft-versus-host disease (GVHD), in which donor immune cells (particularly T lymphocytes) attack recipient organs (1). Corticosteroids remain the primary upfront therapy for GVHD, and steroid-refractory GVHD remains a major cause of morbidity and mortality (2). Second-line treatments for both acute and chronic GVHD lead to cumulative immune suppression and risk for infections. Thus, novel and effective therapies for treatment-refractory GVHD, especially without additive risk of opportunistic infections or organ dysfunction, are urgently needed.

Mesenchymal stromal cells (MSCs) are a regulatory non-hematopoietic immune cell population present in the bone marrow (BM) that can be expanded *ex vivo* to large numbers (3). Based on their ability to suppress the immune system and promote tissue regeneration, MSCs have been evaluated as a treatment for GVHD for nearly two decades (4). Positive clinical trial results have led to the approval of MSCs in Japan for the treatment of GVHD (5), and although US-based trials showed benefit in pediatric patients (6), no benefit was seen in the initial randomized, placebo-controlled trial in adult and pediatric patients (7). Inconsistency in trial results (8) is likely in part due to heterogeneity in cell manufacturing and administration procedures. Three major sources of variability that may impact the clinical efficacy of MSCs have been extensively reviewed: freeze-thawing, replication fitness, and donor source. First, most MSC products are cryopreserved post-expansion and infused immediately post-thaw. However, preclinical data suggest that MSCs are functionally stunned/impaired post-thaw, in comparison to the culture-recovered counterparts (9–11). Second, most MSC products (particularly commercial) have undergone prolonged *ex vivo* expansion, which has been shown to compromise their function. In the setting of acute GVHD treatment, late passage was significantly associated with decreased clinical response and survival (12). Finally, most MSC products have been random donor source, and while MSCs were initially thought to be immune privileged, later studies demonstrated recipient driven immune-mediated rejection (13, 14). While it is not feasible to utilize HLA-matched random donor MSCs, autologous source may be feasible in some settings including GVHD. Importantly, following HCT, the BM MSC compartment remains autologous, and our preclinical data confirm intact phenotype and function of autologous, BM-derived MSCs from patients with GVHD following HCT for hematologic malignancy (15).

By addressing all these limitations, the primary objective of this trial was to evaluate the safety and tolerability of autologous, early-passage, culture-recovered (fresh) MSCs in the setting of treatment-refractory GVHD post-HCT for hematologic malignancy. Thus, within this phase I trial, our primary endpoint was dose-limiting toxicities (DLTs) of an escalating number of weekly MSC infusions, with secondary endpoints examining GVHD response.

## Methods

### Study design

This was a prospective, single‐center, phase I dose-escalation study of autologous MSCs for the treatment of GVHD. The trial followed a standard 3 + 3 design with a fixed MSC dose (2 × 10^6^/kg) and three dose levels with an escalation of the number of doses administered: dose level 1, single infusion; dose level 2, two weekly infusions; dose level 3, four weekly infusions. The protocol was approved by the Emory University Institutional Review Board and the Food and Drug Administration (FDA) (IND 16191) and registered with ClinicalTrials.gov (#NCT02359929). Patients were recruited through the adult and pediatric blood and marrow transplant programs at the Winship Cancer Institute at Emory University and Children’s Healthcare of Atlanta. Written informed consent was obtained prior to enrollment.

### Study population

Patients aged ≥12 years with steroid-refractory or resistant GVHD post-allogeneic HCT for a hematologic malignancy were eligible. GVHD could be grade II–IV acute GVHD requiring systemic therapy and refractory/unresponsive to glucocorticoid (≥1 mg prednisone-equivalent/kg × 1 week); chronic GVHD was extensive and either not improved despite therapy with glucocorticoid (≥0.5 mg prednisone-equivalent/kg/day) and therapeutic doses of a calcineurin inhibitor for ≥4 weeks or worsened within 2 weeks, or overlap syndrome not responding to glucocorticoid treatment (≥1 mg prednisone-equivalent/kg × 1 week). Patients with active fungal infections, evidence of disease relapse, donor chimerism <50%, or oxygen requirement were not eligible to participate. Patients were permitted to receive other systemic immunosuppression per standard of care, including calcineurin inhibitors and steroids. Fifteen patients were enrolled, of whom 11 received some or all planned MSC doses. Four patients were screen failures, and three patients had MSC manufacturing failure, one of whom underwent a second BM collection and MSC expansion, with details shown for an infused product. The trial was closed prior to enrollment of the planned sixth patient on dose level 3, due to no DLTs observed in any of the treated patients (including in dose level 3) and changes in Good Manufacturing Practice (GMP) facility staffing.

### Initial mesenchymal stromal cell manufacturing

BM (1 ml/kg with a maximum of 60 ml) was obtained *via* aspiration under aseptic conditions and then processed for MSC expansion in a class 10000 GMP facility at Emory University Hospital (EUH) per previously published methods (15, 16). In brief, the mononuclear cell (MNC) layer was isolated using Ficoll-Paque™ PREMIUM (MediaTech, Inc., Manassas, VA, USA) density gradient, washed, and resuspended in a complete culture medium (CCM) comprised of HyClone^®^ Minimum Essential Medium (MEM) Alpha Modification (HyClone Laboratories, Logan, UT, USA) with 10% pooled human platelet lysate (phPL; EUH, Atlanta, GA, USA) and gentamicin (prior to P1 only; HyClone Laboratories, Logan, UT, USA). MNCs were then placed into a cellular stack (Corning^®^, Corning, NY, USA) and incubated at 37°C in a 5% CO_2_, humidified environment for 7–10 days (Passage 0 (P0)), with media change at 72 h. MSCs were enzymatically detached and reseeded at approximately 1,000 cells/cm^2^ in culture media containing 10% phPL for an additional 7–10 days (Passage 1 (P1)). If an insufficient number of cells were obtained (per assigned dose level), cells were passaged up to two additional times. Once a sufficient cell number was obtained, cells were collected enzymatically, washed, and counted. Release criteria for cryopreservation included sufficient cell number, viability, identity, negative sterility, endotoxin, and mycoplasma testing (**Supplemental Table 1**). Cells were then resuspended at 10 × 10^6^ MSC/ml in freezing media (5% human serum albumin and 10% dimethyl sulfoxide (DMSO) in CCM), cryopreserved using a programmable control rate freezer, and stored in vapor phase liquid nitrogen until 72 h prior to planned infusion. All cell counting and viability were performed using an Invitrogen Countess™ automated cell counter (Invitrogen, Grand Island, NY, USA).

### Mesenchymal stromal cell culture recovery

Approximately 72 h prior to planned infusion, cells were removed from vapor phase liquid nitrogen, thawed in a 37°C water bath, washed, counted, and seeded onto tissue culture treated plates at a maximum concentration of 50,000 cells/cm^2^ in culture media containing 10% phPL. Media was changed at 24 h, with cells expanded for an additional 1–3 days (median 3 days total, range 2–4). Cells were then collected enzymatically, washed, counted, and resuspended at a concentration of 4 × 10^6^ cells/ml in a solution of Plasma-Lyte A containing 0.05% human serum albumin. Release criteria for infusion included >70% viability and negative gram stain (**Supplemental Table 2**). When release criteria were met, cells were then injected into a standard blood transfusion bag and transported from the manufacturing facility to the site of infusion.

### Mesenchymal stromal cell infusion and patient follow-up

An infusion was performed on either the inpatient unit or outpatient infusion center depending on patient condition. MSCs were infused within 4 h of release using standard blood product tubing and through a central or large bore peripheral intravenous line over 10–20 min by gravity or by a pump. Patients were pre-medicated with acetaminophen, diphenhydramine, and hydrocortisone (or if already on steroids, an equivalent dose). Vital signs were closely monitored during and after (up to 4 h) of the infusion. Targeted adverse events (AEs) were collected on all treated patients for 7 days after each MSC infusion. All serious AEs were collected through study completion. Acute GVHD staging and grading and chronic GVHD scoring were performed per published criteria (17). Patients were followed up for up to 1 year for secondary endpoints.

### Longitudinal analysis of peripheral blood post-mesenchymal stromal cell infusion

Peripheral blood samples were obtained at baseline and then weekly through day 42 from study initiation. Cells were analyzed by flow cytometry for the expression of CD3 (PE-AF594), CD4 (APC-Cy7), CD8 (FITC), CD25 (APC), CD27 (PE), CD69 (PE-Cy7), and FOXP3 (PE; BD Biosciences, San Jose, CA, USA). All samples were run on a Canto II flow cytometer (Beckman Coulter, Indianapolis, IN, USA) and analyzed using FlowJo (BD, Ashland, OR, USA). CD4 and CD8 counts (cells/mm^3^) were calculated using total lymphocyte count from clinical laboratory complete blood counts obtained on the same day.

### Definitions and study endpoints

Systemic reaction was defined as any untoward medical hypersensitivity-like event other than injection site reaction, occurring during or after MSC infusion, which could be at least possibly attributed to the MSC infusion. Acute systemic reactions were defined as those occurring within 2 h of infusion. DLTs were defined as any grade ≥3 adverse reaction that was unexpected or considered attributable to the MSC infusion and occurred within 1 month from the last MSC infusion. Maximum tolerated dose (MTD) was defined as the highest dose level at which at most one of six patients experience a DLT after one cycle, with the immediate higher dose level having at least two patients who experience DLTs.

Overall acute GVHD responses were categorized as complete response (CR), partial response (PR), mixed response (MR), stable disease (SD), or progressive disease (PD), which were defined per published standards. Overall “response” was defined as achieving either CR or PR, while “no response” was defined as achieving MR, SD, or PD. Organ-specific response was classified as improving, stable, progressing, or death. Overall chronic GVHD “response” was defined as a reduction in overall National Institutes of Health (NIH) score at 3 months, without worsening of any specific organ. Organ-specific responses were categorized per NIH criteria as CR, PR, SD, or PD.

The primary endpoint of this phase I trial was safety and tolerability, based on DLTs. Second endpoints included overall and organ-specific acute (at day 29, 4 weeks after the last infusion, 3 months, and 6 months) and chronic (at 3 and 6 months) GVHD responses.

### Statistical analysis

Descriptive statistics were performed on subject clinical and treatment factors, disease response, and flow cytometry data (grouped by dose level). Categorical data are presented as frequency tables and percentages, while continuous data are presented as mean and standard deviation or median and range.

## Results

### Baseline characteristics

Eleven patients with refractory or resistant GVHD received some or all planned autologous MSC doses, with baseline characteristics shown in **Table 1**. The median age at enrollment was 37 years (range, 26–75 years), with most being male (73%) and white (64%). Indications for HCT included leukemia (acute, n = 3; chronic, n = 2), lymphoma (n = 3), myelodysplastic syndrome (n = 2), and myelofibrosis (n = 1), and median time from HCT to first MSC infusion was 2.6 years (range, 0.2–6.5). Most patients received peripheral blood stem cells (PBSC) (73%) from an unrelated donor (URD) (64%). The conditioning regimen was most commonly reduced intensity (n = 6), followed by myeloablative (n = 5); all patients received tacrolimus in combination with mycophenolate mofetil or methotrexate for GVHD prophylaxis.

**Table 1:** Baseline patient, disease, and HCT characteristics.

Study ID	Age (years)	Sex	Race/ethnicity	HCT indication	Time from HCT (years)	Donor	HSC source	Prep regimen	GVHD ppx
EPIC2014-01	35	M	White	ALL	0.5	MUD	BM	Flu/Mel	Tac/MTX
EPIC2014-05	59	M	Native Hawaiian or Other Pacific Islander	AML	0.3	MRD	PBSC	Flu/Mel	Tac/MTX
EPIC2014-06	26	M	White	HL	0.7	MRD	PBSC	Flu/Bu	Tac/MMF
EPIC2014-07	53	M	White	AML	3.4	URD	PBSC	Bu/Cy	Tac/NR
EPIC2014-12	50	M	Black or African American	CTCL	2.6	MUD	PBSC	Flu/Mel	Tac/MTX
EPIC2014-13	34	M	White	MF	4.5	MMUD	PBSC	Bu/Cy	Tac/MTX
EPIC2014-14	55	M	Black or African American	MDS	3.1	MRD	BM	Bu/Cy	Tac/MTX
EPIC2014-15	26	M	White, Hispanic, or Latino	CML	0.4	MUD	PBSC	Bu/Cy	Tac/MTX
EPIC2014-16	28	F	White	HL	6.5	URD	PBSC	FluMel	Tac/MTX
EPIC2014-17	75	F	White	MDS	4.7	MUD	PBSC	FluTBI	Tac/MMF
EPIC2014-18	37	F	Black or African American	CML	0.6	MRD	PBSC	FluMel	Tac/MTX

HCT, hematopoietic cell transplantation; HSC, hematopoietic stem cell; Prep, preparative; GVHD, graft-versus-host disease; ppx, prophylaxis; M, male; F, female; ALL, acute lymphoblastic leukemia; AML, acute myeloid leukemia; HL, Hodgkin lymphoma; CTCL, cutaneous T-cell lymphoma; MF, myelofibrosis; MDS, myelodysplastic syndrome; MUD, matched unrelated donor; MRD, matched related donor; URD, unrelated donor; MMUD, mismatched unrelated donor; BM, bone marrow; PBSC, peripheral blood stem cells; Flu, fludarabine; Mel, melphalan; Bu, busulfan; Cy, cyclophosphamide; TBI, total body irradiation; Tac, tacrolimus; MTX, methotrexate; MMF, mycophenolate mofetil; NR, not reported.

### Mesenchymal stromal cell expansion and culture recovery

From a median starting BM volume of 54 ml (range, 48–60 ml), median starting white blood cell (WBC) count was 1.49e9 (range, 0.43e9–2.85e9), and total nucleated cell (TNC) post-Ficoll was 2.07e8 (range, 0.69e8–11.3e8; **Supplemental Table 3**). Time from initial seeding to P0 was 10 ± 1.8 days (median ± SD), and from P0 to P1, it was 6 ± 1.3 days. Seven products (most commonly those at a higher dose level, with a higher total dose) required additional time in culture, with a median time from P1 to P2 of 7 ± 1.5 days; two of these required additional passage time of 4 and 7 days. Doubling time from P0 to P1 was 1.55 ± 1.66 (median ± SD) days, and from P1 to P2, it was 1.49 ± 0.87 days. Following expansion, MSCs were cryopreserved until approximately 72 h prior to planned infusion, with a median cryopreservation time of 14 days (range, 7–35 days). MSCs were culture recovered for a median of 3 days (range, 2–4) prior to infusion.

### Safety of mesenchymal stromal cell infusion

**Figure 1:**
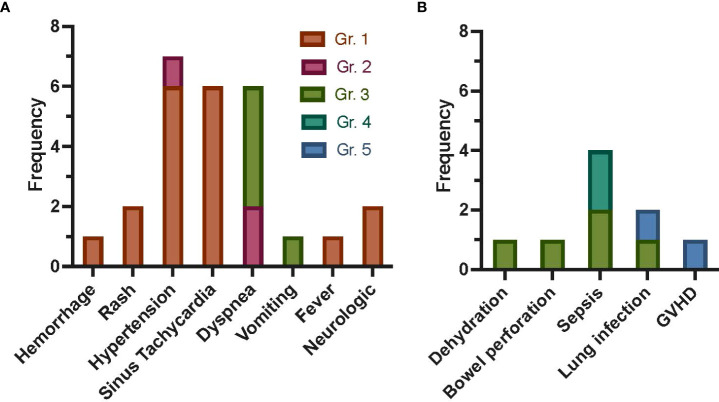
Frequency of AEs following autologous MSC infusion as treatment for refractory GVHD post-HCT for malignant disease. Targeted AEs **(A)** were captured within 7 days of each MSC infusion. Allergic reaction/hypersensitivity, sinus bradycardia, hypotension, rigors/chills, renal, and hypoxia are not shown, as no events occurred. The most common targeted AE was grade 1 or 2 hypertension, with four events occurring in one patient (16). Sinus tachycardia events were all grade 1, with two events occurring in patient 12 and four events occurring in patient 15. All dyspnea events occurred in two patients (n = 2 in patient 7, n = 4 in patient 16). Only three AEs were attributed to study participation: grade 1 rash possibly related, grade 1 rash possibly related, and grade 2 hypertension (30 min after MSC infusion) probably related. SAEs **(B)** were captured for 1 year, with the most common SAE being sepsis (n = 4). Death occurred in two patients (patients 5 and 6, due to GVHD and pneumonia and respiratory failure, respectively). All SAEs were expected and were not attributed (unlikely or definitely not related) to study participation. Gr., grade; GVHD, graft-versus-host disease; AEs, adverse events; MSC, mesenchymal stromal cell; HCT, hematopoietic cell transplantation; SAEs, severe AEs.

Three patients were treated on dose level 1 (single dose), three patients on dose level 2 (weekly × 2 doses), and five patients on dose level 3 (weekly × 4 doses, except one patient who received only two doses due to poor expansion). All doses were 2 × 10^6^ MSC/kg, except for dose 1 in patient 7, who received a dose of 1.27 × 10^6^/kg due to inadequate post-culture recovery. Targeted AEs are shown in **Figure 1**, with the most common being grade 1 or 2 hypertension (n = 4 events occurring in patient 16) followed by sinus tachycardia and dyspnea (each occurring in n = 2 patients). Nine severe AEs (SAEs) occurred (**Figure 1**), including two grade 4 sepsis events and two deaths (grade 5; patient 5 due to multi-organ failure in the setting of GVHD and patient 6 due to pneumonia and respiratory failure, at 115 and 46 days following first MSC infusion, respectively). All SAEs were expected and determined to be unlikely or definitely not related to study participation. Overall, three targeted AEs were attributed to study participation: grade 1 hypertension (probably related), grade 2 hypertension 30 min after MSC infusion (probably related), and grade 1 rash (possibly related). Thus, no DLTs occurred on any of the three dose levels, and an MTD was not reached.

### Graft-versus-host disease characteristics and responses

As shown in **Tables 2**, **3**, all patients had received multiple previous lines of GVHD treatment (range, 3–9), and all were on systemic immunosuppression (with one to three agents) at the time of MSC infusion. In those with a primary indication of acute GVHD at the time of enrollment (patients 1 and 5, both of whom had chronic GVHD at the time of infusion; **Table 2**), responses were seen following a single infusion of MSCs (at 129 and 77 days from GVHD diagnosis, respectively). Patient 1 had skin-only acute GVHD with CR at 6 months and discontinuation of systemic steroids; patient 5 had an MR, with CR of the upper and lower gastrointestinal (LGI) (maximum stage 4) acute GVHD by 3 months but developed new stage 3 liver GVHD. Nonetheless, the systemic steroid dose was able to be decreased in patient 5. Two patients had overlap GVHD as their primary indication for MSC treatment (patients 6 and 15; first MSC infusion at 146 and 122 days from GVHD diagnosis, respectively), with acute GVHD responses seen in one patient (patient 15—on dose level 3) with PR (LGI staging improved to 1 from maximum 2 and decreased systemic steroids). Patient 6 had no response (NR) to a single dose of MSCs with continued stage 4 LGI and eventually death related to complications from GVHD. Thus, 75% of patients with acute GVHD or overlap indication had an overall “response” (at any timepoint) to autologous MSC infusion, and 25% were non-responders.

**Table 2:** Acute GVHD characteristics and response following autologous MSC infusion.

Study ID	Dose level; # doses	Time from GVHD dx (days)	Max grade; stage^1^	Treatment on day 1	Other treatments	Timepoint	Grade	Stage	Overall response	Steroid dose (mg/kg)
EPIC2014-01^2^	1; 1	129	II; 3/0/0/0	Steroid, FK, ECP	ATG	D1	II	2/0/0/0	CR	0.7→0^3^
						D8	I	1/0/0/0		
						D15	I	1/0/0/0		
						D29	I	1/0/0/0		
						D36	I	1/0/0/0		
						3 months	II	3/0/0/0		
						6 months	0	0/0/0/0		
EPIC2014-05	1; 1	77	IV; 0/1/4/0	Steroid, FK, ruxolitinib, ECP	ATG, MMF, infliximab	D1	IV	0/1/4/0	MR	0.9→0.6
						D8	IV	0/1/4/0		
						D15	II	0/0/1/0		
						D29	IV	0/0/4/0		
						D36	I	0/1/0/0		
						D42	IV	0/1/4/0		
						2 months	I	0/1/0/0		
						3 months	III	0/0/0/3		
EPIC2014-06^4^	1; 1	146	IV; 0/1/4/0	Steroid, FK	Sirolimus, MMF, ECP	D1	IV	0/0/4/0	NR	1.1→0.7
						D8	IV	0/1/4/0		
						D15	IV	0/0/4/0		
						D29	IV	0/0/4/0		
						D36	IV	0/1/4/0		
EPIC2014-15	3; 4	122	III; 0/0/2/0	Steroid, FK	Remicade, Jakafi	D1	II	0/0/1/0	PR	1.0→0
						D8	II	0/0/1/0		
						D15	II	0/0/1/0		
						D29	II	0/0/1/0		
						D36	III	0/0/2/0		
						2 months	III	0/0/2/0		
						3 months	III	0/0/2/0		
						6 months	II	0/0/1/0		
						12 months	II	0/0/1/0		

GVHD, graft-versus-host disease; MSC, mesenchymal stromal cells; Max, maximum; FK, tacrolimus; ECP, extracorporeal photopheresis; ATG, anti-thymocyte globulin; MMF, mycophenolate mofetil; CR, complete response; NR, no response; PR, partial response; UGI, upper gastrointestinal; LGI, lower GI.

(1)Stage reported as skin/UGI/LGI/liver.

(2)Chronic GVHD diagnosed on 2/24/2015. Skin GVHD developed chronic features during the study.

(3)Steroids weaned off within a few days of first MSC infusion. (4)Overlap GVHD with acute phenotype.

**Table 3:** Chronic GVHD characteristics and response following autologous MSC infusion.

Study ID	Dose level	# Doses	Time from GVHD dx (years)	Baseline severity	Treatment on day 1	Other treatments	Severity at last f/u	Overall response^1^	Steroid dose (mg/kg)
EPIC2014-07	2	2	2.1	Severe	Steroid, FK^2^	Sirolimus, MMF, ruxolitinib, ECP, imatinib, MTX, rituximab	Severe	PR	0.3→0.3
EPIC2014-12	2	2	0.2^3^	Mod	Steroid	FK, ruxolitinib	Mod	PR	0.5→0.2
EPIC2014-13	2	2	0.5^4^	Severe	FK^5^	Steroid, ruxolitinib, ibrutinib, ECP	Severe	PD	0→0
EPIC2014-14	3	4	1.7	Severe	Steroid, FK, ruxolitinib	N/A	Severe	SD	0.1→0.1
EPIC2014-16	3	4	6.2	Severe	Steroid, sirolimus, ibrutinib^6^	FK, ECP	Severe	PR	0.6→0.4
EPIC2014-17^7^	3	2	4.4	Severe	Ruxolitinib, MMF	Steroid, rituximab, FK, MMF, imatinib, sirolimus, ECP, ruxolitinib	Mod	PR	0→0
EPIC2014-18^8^	3	4	0.2	Mild	Steroid, FK, ruxolitinib	N/A	Mild	PR	0.2→0

GVHD, graft-versus-host disease; MSC, mesenchymal stromal cells; dx, diagnosis; f/u, follow-up; mod, moderate; FK, tacrolimus; MMF, mycophenolate mofetil; ECP, extracorporeal photopheresis; MTX, methotrexate; PR, partial response; PD, progressive disease; SD, stable disease; EGD, esophagogastroduodenoscopy; UGI, upper gastrointestinal; LGI, lower GI.

(1)Overall response assessed at 3 months following first MSC infusion.

(2)Ibrutinib started 3 months following first MSC infusion.

(3)Date of diagnostic EGD, prior history of skin and nail chronic GVHD.

(4)Overlap GVHD diagnosed on 4/30/2015.

(5)Jakafi started at 9 months following first MSC infusion.

(6)Ruxolitinib started 5 months following first MSC infusion and ibrutinib discontinued.

(7)History of acute skin only GVHD, quiescent at study enrollment.

(8)At enrollment, patient had acute GVHD (stage 1 UGI, 2 LGI); at treatment, patient had only chronic GVHD.

**Figure 2:**
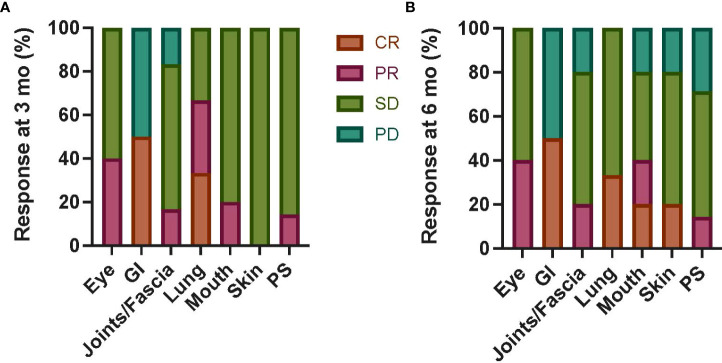
Chronic GVHD organ-specific responses following autologous MSC infusion. Organ-specific responses were assessed at 3 months **(A)** and 6 months **(B)** from first MSC infusion in six patients with primary indication of chronic GVHD regardless of dose level. CR was seen in a subset of patients with GI and lung involvement at both timepoints; otherwise, most patients had PR or SD in all organs. The proportion of patients with PD was highest in the GI system (50% at both 3 and 6 months), with a smaller proportion in joints/fascia, mouth (3 months only), and PS (3 months only) systems. Mo, months; GI, gastrointestinal; PS, performance score; CR, complete response; PR, partial response; SD, stable disease; PD, progressive disease; GVHD, graft-versus-host disease; MSC, mesenchymal stromal cell.

 In those with chronic GVHD as the primary indication for MSC (n = 7; **Table 3** and **Supplementary Table 4**), an overall response at 3 months was partial in five, stable in one, and progressive in one (with an improved total score but increased GI and joint scores). In GI and lung organ systems, 50% and 33% of patients, respectively, had a CR at 3 and 6 months (**Figure 2**), with lung responses otherwise PR or SD, but progressive GI disease in remaining patients. Otherwise, most organ-specific responses were PR or SD in most patients. PD was seen in a smaller proportion of patients with joint/fascia, mouth (6 months only), skin (6 months only), or PS (6 months only) systems involved.

### Longitudinal immune profile

**Figure 3:**
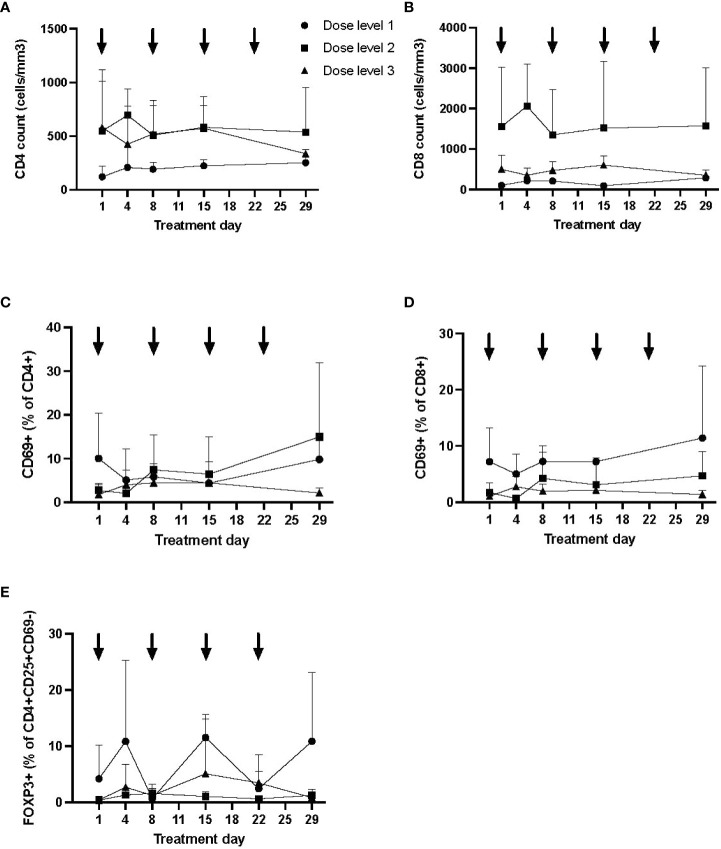
Longitudinal immune profile following autologous MSC infusion. Flow cytometry was performed to evaluate lymphocyte subsets (CD3+CD4+ and CD3+CD8+; **A**, **B**), T-cell activation (CD69+; **C**, **D**), and regulatory T cells **(E)** longitudinally. Data (mean, SD) are shown by dose level, with timing of MSC infusion designated by arrows. The proportion of activated T cells was lowest at day 29 in dose level 3, although T-cell activation was higher at baseline in dose level 1. No significant differences were otherwise seen by dose level in T-cell counts or proportion of regulatory T cells. MSC, mesenchymal stromal cell; SD, standard deviation.

CD4 and CD8 lymphocyte counts and percentage of activated (CD69+) subsets are shown in **Figure 3** (mean and SD), broken down by dose level. While CD4 and CD8 counts did not appear to differ between dose levels or over time, the percentage of activated CD4 and CD8 lymphocytes was the lowest at day 29 in those treated on dose level 3 (noting that dose level 1 patients had the highest percentage at baseline). No appreciable difference was detected in the percentage of regulatory T cells (FOXP3+ cells within CD3+CD4+CD8−CD25+CD69−) between dose levels (**Figure 3**).

## Discussion

In this phase I dose-escalation trial of autologous MSCs for treatment-refractory GVHD following HCT for hematologic malignancy, no DLTs were seen, and an MTD was not reached, with the highest dose level being weekly infusions of 2 × 10^6^/kg × 4 doses. Secondary endpoints included clinical response, and while limited by the small sample size and heterogeneity of GVHD, three of four patients with acute GVHD (or overlap with acute component) had objective responses ranging from partial to complete. Importantly, this trial demonstrates the feasibility of using autologous and culture-recovered MSCs to treat GVHD.

To our knowledge, this is the first clinical trial to evaluate autologous, fresh (culture recovered), and early-passage MSCs in the treatment of GVHD to overcome barriers in donor source and culture conditions, which may have impacted the efficacy of MSCs in previous trials. Given that most clinical trials have used cryopreserved, freshly thawed MSCs, it was important to first verify the safety of this approach. Additionally, our product likely costs considerably less than commercial MSC products such as Remestemcel-L, which has been reported to cost $195,000 in Japan. Our safety findings are consistent with previous studies, including a meta-analysis restricted to randomized controlled trials of MSCs for a variety of inflammatory conditions (18); while transient fever was associated with MSC infusion in this meta-analysis, this may have been abrogated in our population given steroid pre-treatment and concurrent immunosuppression for treatment of GVHD. Additionally, our safety and feasibility results are consistent with our previously published phase I dose-escalation trial of autologous and culture-recovered MSCs as treatment for refractory Crohn’s disease, where no DLTs were seen (16).

This approach could further improve efficacy—given our phase I design, small sample size, and heterogeneity in patients (including concurrent GVHD treatment), we were limited in our ability to detect clinical responses to our MSC product. Nonetheless, our data suggest that an acute GVHD phenotype may be more responsive to MSC treatment, with the only acute GVHD patient having no response to MSCs having been on >1 mg/kg/day of steroids. Compared to the efficacy results of other MSC trials, our efficacy results in acute GVHD patients compare favorably and in chronic GVHD patients are comparable, while providing more detailed outcome data (with longitudinal organ scoring). As recently reviewed by Kelly et al. (4), MSCs have been evaluated for the treatment of GVHD in nearly 60 ongoing or completed clinical trials, primarily for steroid-refractory acute GVHD and in a single-arm, small, phase I or II clinical trials. Heterogeneity in MSC product (including starting product, passage, and dose) and definitions (including treatment-refractory GVHD and response) limit the ability to assess responses across trials. Nonetheless, in the treatment of steroid-refractory acute GVHD, responses have been mixed, with day 28 CR ranging from 8% to 75% and higher OR ranging from 42% to 100%; notably, many trials did not specify the timing of response assessment. In the two studies with a control group, responses were higher in those treated with MSCs; in those with two dose levels (n = 2), the dose response was mixed. There are limited data on MSCs as a first-line treatment for acute GVHD, with both trials showing paradoxical lower responses in those receiving a higher MSC dose. Fewer trials of MSCs for chronic GVHD treatment have been performed, all small (n = 1–14 patients) and single arm. Responses have also been mixed, with CR at day 28 ranging from 0% to 40% and OR ranging from 0% to 80%; in one trial reporting responses at 1 year, CR and OR were 80%. Across all studies (acute and chronic GVHD), overall survival post-MSC treatment was also mixed, ranging from 0% to 100%. Potentially reduced potency in post-thawed products and use of late-passage MSCs may in part explain variability in observed responses. Thus, methods to improve MSC potency and standardized product potency assays may assist in comparisons across clinical trials (19). In our study, we did not observe any changes in lymphocyte count, lymphocyte activation, or regulatory T cells in the first month to correlate with treatment exposure or response. Future trials should emphasize the performance of correlative analyses to identify pharmacodynamic evidence of MSC activity and predictors of response.

Our efficacy data also appear similar to other second-line treatments for treatment-refractory GVHD, particularly for acute GVHD. Ruxolitinib is the only agent currently approved by the FDA for the treatment of steroid-refractory acute GVHD, based on clinical trial data with CR ranging from 27% to 34% and OR ranging from 55% to 62% at day 28 (20, 21). In chronic GVHD, three agents are now approved for the treatment of refractory disease, ibrutinib, belumosudil, and ruxolitinib, with best OR ranging from 67% to 76% and CR ranging from 5% to 21% (22). Steroids remain the pillar of upfront therapy for GVHD but often lead to significant morbidity including likely impairment in epithelial healing, which can further complicate the clinical picture of GI GVHD (23). The cumulative effect of added immune suppression can lead to opportunistic infections and further toxicity. Alternative approaches focused on promoting tolerance and tissue healing may offer efficacy without additive risk for infection. In addition to MSCs, another example is the use of urinary-derived human chorionic gonadotropin/epidermal growth factor (uhCG/EGF) for the treatment of severe acute GVHD (24, 25).

The use of autologous MSCs in the setting of GVHD is limited by the timeline required for *ex vivo* expansion, especially in the treatment of acute GVHD, where the escalation of therapy is needed after 1 week if refractory to steroids. An autologous source of MSCs for chronic GVHD treatment is likely not feasible, as treatment beyond four doses is likely required, and expansion for >4 doses while maintaining early passage is not achievable. The use of early passage and culture-recovered MSCs, regardless of BM source, is likely to offer improved potency over freshly thawed and multiply passaged MSCs, though this requires further evidence from future clinical trials with associated biologic correlates. Informed by our phase I trial results and with a continued goal to improve the *in vivo* efficacy of MSC infusion, we have thus launched a clinical trial of third-party cryopreserved MSCs to evaluate interferon-gamma priming during culture recovery, based on preclinical studies demonstrating enhanced potency (NCT04328714) (10, 26, 27).

In conclusion, we determined that autologous MSCs given weekly for four doses are safe in the setting of treatment-refractory GVHD post-HCT for hematologic malignancy. Culture recovery may reverse the deleterious impact of cryopreservation and thawing on MSC potency, and thus the safety signal in this trial supports this manufacturing approach in now ongoing and future GVHD trials.

## Data availability statement

The original contributions presented in the study are included in the article/**Supplementary Material**. Further inquiries can be directed to the corresponding author.

## Ethics statement

The studies involving human participants were reviewed and approved by Emory University IRB. Written informed consent to participate in this study was provided by the participants’ legal guardian/next of kin.

## Author contributions

AL, JG, EW, and MQ designed the trial. CG, AL, DK, PD, RC, JG, EW, and MQ participated in the study execution and data collection. ES, CG, PD, and MQ analyzed the data. All authors interpreted the data, critically reviewed the manuscript, and provided final approval for submission. All authors agree to be accountable for all aspects of the work, ensuring the accuracy and integrity of the publication.

## Funding

Supported by: National Center for Advancing Translational Sciences (KL2TR000455) (MQ) and CURE Childhood Cancer (MQ).

## Acknowledgments

The authors would like to acknowledge the contributions of Ian Copland, PhD (posthumous) and Marco Garcia.

## Conflict of interest

The authors declare that the research was conducted in the absence of any commercial or financial relationships that could be construed as a potential conflict of interest.

## Publisher’s note

All claims expressed in this article are solely those of the authors and do not necessarily represent those of their affiliated organizations, or those of the publisher, the editors and the reviewers. Any product that may be evaluated in this article, or claim that may be made by its manufacturer, is not guaranteed or endorsed by the publisher.
