# Supplementary material for: Safety of autologous freshly expanded mesenchymal stromal cells for the treatment of graft-versus-host disease
Source: Front Immunol. 2022 Sep 14;13:959658. doi: 10.3389/fimmu.2022.959658 (PMC9515357; doi:10.3389/fimmu.2022.959658)
Supplement: Supplementary file 1 [file DataSheet_1.docx]

| **Supplemental Table 1: Release Criteria of cryopreserved MSC product for potential infusion.** | | | |
| --- | --- | --- | --- |
| **Test** | **Method** | **Specifications** | **Testing Frequency** |
| ***Product Characteristics*** | | | |
| Cell number | Automated cell counter | 2X required cell dose ±15% | Prior to cryopreservation |
| Viability | Trypan Blue exclusion | 85%±15% | Prior to cryopreservation |
| ***Product Identity and Purity*** | | | |
| Identity | Flow cytometry | >85% CD90, CD73, CD105 | Post-cryopreservation |
| Impurities | Flow cytometry | <3% CD45 | Post-cryopreservation |
| ***Process Related Impurities*** | | | |
| Endotoxin | LAL Clot | < 0.1 EU/mL | At BM receipt, at first passage, post-cryopreservation. |
| Sterility | BacT Alert | No growth (5 day) | At BM receipt, at first passage, post-cryopreservation. |
| Mycoplasma | MycoAlert® Assay | None detected | At BM receipt, at first passage, post-cryopreservation. |

MSC denotes mesenchymal stromal cells; LAL, Limulus Amebocyte Lysate; and BM, bone marrow.

| **Supplemental Table 2: Release Criteria for MSC product at time of Infusion** | | | |
| --- | --- | --- | --- |
| **Test** | **Method** | **Specifications** | **Testing Frequency** |
| ***Product Characteristics*** | | | |
| Cell number | Automated cell counter | Required cell dose ±15% | Prior to infusion |
| Viability | Trypan Blue exclusion | 85%±15% | Prior to infusion |
| ***Product Identity and Purity*** | | | |
| Impurities | Flow cytometry | <3% CD45 | Prior to infusion |
| ***Process Related Impurities*** | | | |
| Gram Stat | Microscopy | No vegetative growth | Prior to infusion |

MSC denotes mesenchymal stromal cells.

| **Supplemental Table 3: Summary of fresh MSCs expanded from BM derived from patients with refractory GVHD** | | | | | | | | | | |
| --- | --- | --- | --- | --- | --- | --- | --- | --- | --- | --- |
| **Study ID** | **BM volume (mL)** | **WBC count pre-Nicoll** | **TNC post-Ficoll** | **P0 MSC count^1^** | **MSC reseeded** | **P1 MSC count^2^** | **MSC reseeded** | **P2 MSC count^3^** | **Doubling time (d; P0→P1)** | **Doubling time (d; P1→P2)** |
| EPIC2014-01 | 60 | 1.49E+09 | 6.93E+07 | 2.70E+06 | 2.70E+06 | 7.40E+07 | 1.6E+05 | 1.74E+08 | 1.47 | 0.69 |
| EPIC2014-05 | 60 | 6.60E+08 | 1.74E+08 | 1.31E+07 | 1.31E+07 | 2.41E+08 | N/A | N/A | 1.67 | N/A |
| EPIC2014-06 | 60 | 1.73E+09 | 8.04E+08 | 5.00E+07 | 5.00E+07 | 5.70E+08 | N/A | N/A | 1.14 | N/A |
| EPIC2014-07 | 53 | 1.85E+09 | 1.69E+08 | 3.28E+06 | 3.28E+06 | 3.26E+07 | 3.26E+07 | 3.48E+08 | 2.42 | 2.05 |
| EPIC2014-12 | 54 | 8.50E+08 | 2.41E+08 | 6.65E+06 | 6.65E+06 | 6.80E+07 | 6.80E+07 | 1.11E+09 | 1.19 | 1.49 |
| EPIC2014-13^4^ | 53 | 1.00E+09 | 2.93E+08 | 4.76E+05 | 4.76E+05 | 1.07E+07 | 9.0E+06 | 3.98E+08 | 1.56 | 1.34 |
| EPIC2014-14^4^ | 48 | 1.49E+09 | 1.67E+08 | NR | NR | 2.78E+07 | 2.78E+07 | 2.40E+08 | N/A | 2.25 |
| EPIC2014-15 | 57 | 2.00E+09 | 1.13E+09 | 1.26E+08 | 1.26E+08 | 7.95E+08 | N/A | N/A | 2.26 | N/A |
| EPIC2014-16 | 54 | 4.32E+08 | NR | 3.04E+06 | 3.04E+06 | 7.48E+07 | 7.48E+07 | 6.50E+08 | 1.52 | 1.28 |
| EPIC2014-17 | 54 | 8.64E+08 | NR | 1.36E+07 | 1.36E+07 | 2.30E+07 | 2.30E+07 | 1.47E+08 | 6.60 | 3.37 |
| EPIC2014-18 | 50 | 2.85E+09 | NR | 3.64E+07 | 2.25E+06 | 7.77E+08 | N/A | N/A | 0.83 | N/A |
| Median±SD | 54 | 1.49E+09 | 2.07E+08 | - | - | - | - | - | 1.55±1.66 | 1.49±0.87 |
| Min | 48 | 4.32E+08 | 6.93E+07 | - | - | - | - | - | 0.83 | 0.69 |
| Max | 60 | 2.85E+09 | 1.13E+09 | - | - | - | - | - | 6.60 | 3.37 |

^1^Median±SD time in culture from initial seeding to P0 was 10±1.8 days

^2^Median±SD time in culture from initial P0 to P1 was 6±1.3 days

^3^Median±SD time in culture from initial P1 to P2 was 7±1.5 days

^4^Pts 13 and 14 required P3 (for 4 and 7 days), with 2.0E+08 and 2.4E+08 cells seeded and 11.5E+08 and 5.3E+08 MSCs counted at P3, respectively.

MSCs denotes mesenchymal stromal cells; BM, bone marrow; GVHD, graft-versus-host disease; mL, milliliters; WBC, white blood cell; TNC, total nucleated cells; P0, passage 0; P1, passage 1; P2, passage 2; d, days; NR, not reported; N/A, not applicable; SD, standard deviation; Min, minimum; and Max, maximum.

| **Supplemental Table 4: Chronic GVHD organ specific involvement at screening and longitudinal follow up after autologous MSC infusion** | | | | | | | | | | | | | |
| --- | --- | --- | --- | --- | --- | --- | --- | --- | --- | --- | --- | --- | --- |
| **Study ID** | **Timepoint** | **Eye** | **GU^1^** | **GI** | **Joints/Fascia** | **Liver** | **Lung** | **Mouth** | **Skin** | **PS** | **Other** | **Total** | **Severity** |
| **EPIC2014-07** | D1 | 2 | 0 | 0 | 2 | 0 | 3 | 1 | 2 | 1 | 0 | 11 | Severe |
|  | D29 | 1 | 0 | 0 | 2 | 0 | 3 | 1 | 2 | 1 | 0 | 10 | Severe |
|  | D42 | 1 | 0 | 0 | 2 | 0 | 2 | 1 | 2 | 1 | 0 | 9 | Severe |
|  | 2 mo | 1 | 0 | 0 | 2 | 0 | 2 | 1 | 2 | 1 | 0 | 9 | Severe |
|  | 3 mo^2^ | 1 | 0 | 0 | 2 | 0 | 2 | 1 | 2 | 1 | 0 | 9 | Severe |
|  | 6 mo | 1 | 0 | 0 | 2 | 0 | 3 | 2 | 2 | 1 | 0 | 11 | Severe |
|  | 9 mo | 1 | 0 | 0 | 2 | 0 | 3 | 2 | 2 | 1 | 0 | 11 | Severe |
| **EPIC2014-12** | D1 | 0 | 0 | 1 | 1 | 0 | 0 | 2 | 0 | 1 | 0 | 5 | Mod |
|  | D29 | 0 | 0 | 0 | 1 | 0 | 0 | 1 | 0 | 1 | 0 | 3 | Mod |
|  | 3 mo | 0 | 0 | 0 | 1 | 0 | 0 | 1 | 0 | 1 | 0 | 3 | Mod |
|  | 6 mo | 0 | 0 | 0 | 1 | 0 | 0 | 1 | 0 | 1 | 0 | 3 | Mod |
|  | 9 mo | 0 | 0 | 0 | 2 | 0 | 0 | 1 | 2 | 2 | 0 | 7 | Mod |
|  | 12 mo | 0 | 0 | 0 | 2 | 0 | 0 | 1 | 2 | 2 | 4 | 11 | Mod |
| **EPIC2014-13** | D1 | 3 | 0 | 0 | 2 | 0 | 0 | 1 | 3 | 2 | 1 | 12 | Severe |
|  | D29 | 2 | 0 | 0 | 2 | 1 | 0 | 1 | 3 | 2 | 0 | 11 | Severe |
|  | 2 mo | 1 | 0 | NR | 2 | 0 | NR | NR | 3 | 3 | 3 | 12 | Severe |
|  | 3 mo | 1 | 0 | 2 | 3 | 0 | 0 | 1 | 3 | 1 | 0 | 11 | Severe |
|  | 6 mo | 1 | 0 | 2 | 2 | 0 | 0 | 1 | 3 | 1 | 0 | 10 | Severe |
|  | 9 mo^3^ | 1 | 0 | 2 | 3 | 0 | 0 | 1 | 3 | 2 | 0 | 12 | Severe |
|  | 12 mo | 1 | 0 | 3 | 3 | 0 | 0 | 1 | 3 | 2 | 0 | 12 | Severe |
| **EPIC2014-14** | D1 | 2 | 0 | 0 | 2 | 0 | 1 | 0 | 3 | 1 | 0 | 9 | Severe |
|  | D29 | 2 | 0 | 0 | 2 | 0 | 0 | 0 | 3 | 1 | 0 | 8 | Severe |
|  | 2 mo | 2 | 0 | 0 | 2 | 0 | 0 | 0 | 3 | 1 | 0 | 8 | Severe |
|  | 3 mo | 2 | 0 | 0 | 2 | 0 | 0 | 0 | 3 | 1 | 0 | 8 | Severe |
|  | 6 mo | 2 | 0 | 0 | 2 | 0 | 0 | 0 | 3 | 1 | 0 | 8 | Severe |
|  | 12 mo | 2 | 0 | 0 | 2 | 0 | 1 | 1 | 3 | 1 | 0 | 10 | Severe |
| **EPIC2014-16** | D1 | 2 | 0 | 0 | 2 | 0 | 3 | 1 | 3 | 1 | 0 | 12 | Severe |
|  | D29 | 2 | 0 | 0 | 2 | 0 | 3 | 1 | 3 | 1 | 0 | 12 | Severe |
|  | 2 mo | 2 | 0 | 0 | 2 | 0 | 3 | 1 | 3 | 1 | 0 | 12 | Severe |
|  | 3 mo | 2 | 0 | 0 | 2 | 0 | 3 | 1 | 3 | 1 | 0 | 12 | Severe |
|  | 6 mo^4^ | 2 | 0 | 0 | 2 | 0 | 3 | 1 | 0 | 2 | 0 | 10 | Severe |
|  | 9 mo | - | - | - | - | - | - | - | - | - | - | - | - |
|  | 12 mo | 2 | 0 | 0 | 2 | 0 | 3 | 1 | 0 | 2 | 0 | 10 | Severe |
| **EPIC2014-17** | D1 | 1 | 0 | 0 | 2 | 0 | 0 | 0 | 3 | 1 | 0 | 7 | Severe |
|  | D29 | 1 | 0 | 0 | 2 | 0 | 0 | 0 | 3 | 1 | 0 | 7 | Severe |
|  | 2 mo | 1 | 0 | 0 | 1 | 0 | 0 | 0 | 3 | 1 | 0 | 6 | Severe |
|  | 3 mo | 1 | 0 | 0 | 1 | 0 | 0 | 0 | 3 | 1 | 0 | 6 | Severe |
|  | 4 mo | 1 | 0 | 0 | 2 | 0 | 0 | 0 | 3 | 1 | 0 | 7 | Severe |
|  | 5 mo | 1 | 0 | 0 | 2 | 0 | 0 | 0 | 3 | 1 | 0 | 7 | Severe |
|  | 6 mo | 1 | 0 | 0 | 1 | 0 | 0 | 0 | 3 | 1 | 0 | 6 | Severe |
|  | 9 mo | 1 | 0 | 0 | 1 | 0 | 0 | 0 | 3 | 1 | 0 | 6 | Severe |
|  | 12 mo | 0 | 0 | 1 | 2 | 0 | 0 | 0 | 3 | 1 | 0 | 8 | Severe |
| **EPIC2014-18** | D1 | 0 | 0 | 0 | 0 | 0 | 0 | 1 | 0 | 1 | 0 | 2 | Mild |
|  | D29 | 0 | 0 | 0 | 0 | 0 | 0 | 1 | 0 | 1 | 0 | 2 | Mild |
|  | 2 mo | 0 | 0 | 0 | 0 | 0 | 0 | 1 | 0 | 1 | 0 | 2 | Mild |
|  | 3 mo | 0 | 0 | 0 | 0 | 0 | 0 | 1 | 0 | 1 | 0 | 2 | Mild |
|  | 4 mo | 0 | 0 | 0 | 0 | 0 | 0 | 1 | 0 | 1 | 0 | 2 | Mild |
|  | 6 mo | 0 | 0 | 0 | 0 | 0 | 0 | 0 | 0 | 1 | 0 | 1 | Mild |
|  | 9 mo | 0 | 0 | 0 | 0 | 0 | 0 | 0 | 0 | 1 | 0 | 1 | Mild |
|  | 12 mo | 0 | 0 | 0 | 0 | 0 | 0 | 0 | 0 | 1 | 0 | 1 | Mild |
| **Organ-specific responses** | | | | | | | | | | | | | |
| **Response** | **Timepoint** | **Eye** | **GU^1^** | **GI** | **Joints/Fascia** | **Liver** | **Lung** | **Mouth** | **Skin** | **PS** | **Other** | - | - |
| CR | 3 mo | 0 | N/A | 1 | 0 | N/A | 1 | 0 | 0 | 0 | N/A | - | - |
| PR |  | 2 | N/A | 0 | 1 | N/A | 1 | 1 | 0 | 0 | N/A | - | - |
| SD |  | 3 | N/A | 0 | 4 | N/A | 1 | 4 | 5 | 1 | N/A | - | - |
| PD |  | 0 | N/A | 1 | 1 | N/A | 0 | 0 | 0 | 6 | N/A | - | - |
| CR | 6 mo | 0 | N/A | 1 | 0 | N/A | 1 | 1 | 1 | 0 | N/A | - | - |
| PR |  | 2 | N/A | 0 | 1 | N/A | 0 | 1 | 0 | 1 | N/A | - | - |
| SD |  | 3 | N/A | 0 | 4 | N/A | 2 | 2 | 4 | 4 | N/A | - | - |
| PD |  | 0 | N/A | 1 | 1 | N/A | 0 | 1 | 1 | 2 | N/A | - | - |

^1^GU examination not performed in most patients.

^2^Ibrutinib started at 3 months following 1^st^ MSC infusion.

^3^Jakafi started at 9 months following 1^st^ MSC infusion.

^4^Ruxolitinib started at 5 months following 1^st^ MSC infusion and ibrutinib discontinued.

GVHD denotes graft-versus-host disease; MSC, mesenchymal stromal cell; GU, genitourinary; PS, performance score; D, day; mo, months; NR, not reported; Mod, moderate; and N/A, not applicable.
